# Myasthenia gravis coexisting with *HINT1*-related motor axonal neuropathy without neuromyotonia: a case report

**DOI:** 10.1186/s12883-022-02690-6

**Published:** 2022-05-03

**Authors:** Jia Fang, Hui Huang, Qiang Lei, Yingying Luo, Zhengchu Tang, Xiaoliu Shi, Jian Guang Tang

**Affiliations:** 1 Department of Neurology, The Second Xiangya Hospital, Central South University, Changsha, Hunan China; 2 Department of Medical Genetics, The Second Xiangya Hospital, Central South University, Changsha, Hunan China

**Keywords:** Case report, Myasthenia gravis, HINT1, Novel, Motor axonal neuropathy, Next-generation sequencing

## Abstract

**Background:**

*HINT1* mutations cause an autosomal recessive axonal neuropathy with neuromyotonia. This is a first case report of coexistence of myasthenia gravis (MG) and *HINT1*-related motor axonal neuropathy without neuromyotonia.

**Case presentation:**

A 32-year-old woman presented with recurrent ptosis for 8 years, diplopia for 2 years and limb weakness for 1 year and a half. Neostigmine test, elevated AChR antibody level and positive repetitive nerve stimulation supported the diagnosis of MG. Electroneurography (ENG) and electromyography (EMG) examinations revealed a motor axonal neuropathy without neuromyotonic or myokymic discharges. Next-generation sequencing and Sanger sequencing were performed to identify the gene responsible for suspected hereditary neuropathy. Genetic testing for a *HINT1* mutation was performed and revealed a homozygous mutation at c.278G>T (p. G93V). The patient was treated with pyridostigmine, oral prednisolone and azathioprine. Her ptosis and diplopia have significantly improved at 6-month follow-up.

**Conclusions:**

Concurrence of MG and hereditary motor axonal neuropathy without neuromyotonia is quite rare. Detection of ptosis with or without ophthalmoplegia, distribution of limb weakness, and reflex can help in recognizing the combination of MG and peripheral neuropathy. Early diagnosis is important for initial treatment and prognosis. The novel homozygous variant c.278G>T(p.G93V) contributes to the pathogenic variants spectrum of the HINT1 gene.

## Background

Myasthenia gravis (MG) is an autoimmune antibody-mediated disorder of neuromuscular synaptic transmission [1]. MG is clinically characterized by fluctuating muscle weakness with oculobulbar muscles affected earlier and more frequently than other muscles [1]. Muscle weakness usually shows dramatic improvement on cholinesterase inhibitors. Electrophysiological studies are characterized by decrement in compound muscle action potential (CMAP) amplitude in response to low-frequency repetitive nerve stimulation (RNS).

In 2012, pathogenic mutations in the gene encoding the histidine triad nucleotide binding protein 1 (HINT1) were identified by Zimoń et al. in autosomal recessive motor predominant axonal neuropathy with neuromyotonia [2]. *HINT1* mutations may account for 11% of all inherited neuropathies with autosomal recessive inheritance and for 80% of individuals with axonal neuropathy having the clinical hallmark of neuromyotonia. *HINT1* neuropathy has a worldwide distribution and is particularly prevalent in European countries [3]. Recently it has been reported in Chinese population [4, 5].

In the present study, we reported the first case of ocular MG, whose diagnosis was challenged by the coexistence of *HINT1*-related hereditary axonal neuropathy without clinical or electrophysiological features of neuromyotonia.

## Case presentation

A 32-year-old female was admitted to hospital due to recurrent bilateral ptosis for 8 years, diplopia for 2 years and limb weakness for 1 year and a half. Eight years ago, she developed ptosis. After taking mecobalamin orally for a few months, her symptoms completely recovered. Two years ago, ptosis recurred along with diplopia, and half a year later, her condition was aggravated by limb weakness with the distal muscles more severely affected than the proximal muscles. Since then, she couldn’t take care of herself independently, and couldn’t walk on a level road without support. Fluctuations in muscle strength were not pronounced. She had normal motor development milestones but began to notice poor performance in sports since school age, and during her adult life. She can only do some indoor housework, but not the outdoor heavy farm work. During the course of the disease, no neurodevelopmental abnormalities or psychiatric symptoms were observed. The patient was born from consanguineous parents, and she has a negative family history of neurological or neuromuscular disease.

Her physical examination on admission revealed bilateral ptosis, complete external ophthalmoplegia, and diplopia. Muscle strength was 2/5 for finger/wrist extension and ankle dorsiflexion/plantar flexion, 3/5 for wrist/finger bending, while proximal muscle strength was 4/5 for upper limbs and lower limbs (grades 0–5 on the Medical Research Council Scale). The tendon reflex of both upper limbs was normal and symmetrical. The knee reflex and Achilles tendon reflex were absent, and the plantar reflexes were flexor. There were atrophy of the distal muscles of upper and lower limbs, per cavus, claw-like hands, and a steppage gait. Sensory examinations were normal. No signs of neuromyotonia were observed.

**Fig. 1:**
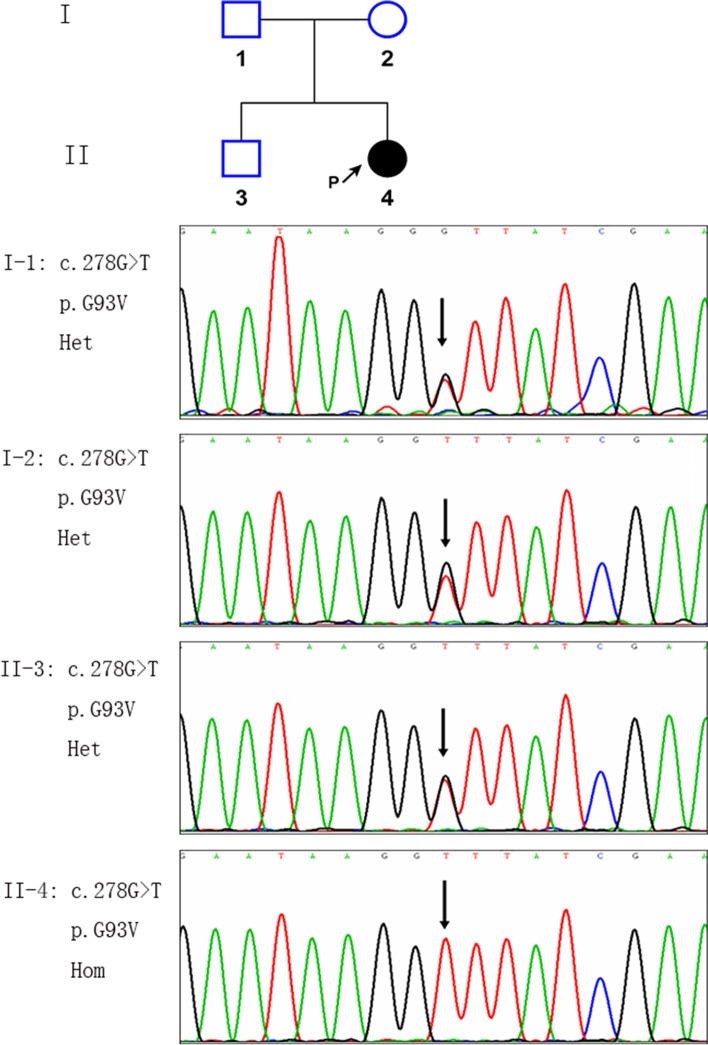
A homozygous mutation of the c.278G > T(p.G93V) of the HINT1 gene was identified in the proband and marked with arrow. The same heterozygous variant was found in the patient’s father, mother, and brother

Intramuscular injection of neostigmine 1 mg significantly improved her ptosis and diplopia. The acetylcholine receptor (AChR) antibody was elevated (11.793 nmol/L, normal < 0.5 nmol/L). Immunofixation electrophoresis in blood and urine was normal. Thyroid function was normal. Computed tomography of the chest showed no parenchymal abnormalities. The result of lumbar puncture cerebrospinal fluid examination was normal. Brain and spine MRI were not done, since there were no neuropsychiatric symptoms. Electromyographic studies revealed a chronic motor axonal neuropathy, without neuromyotonic or myokymic discharges (Table 1). Electromyography (EMG) showed complex repetitive discharges (CRD) in the right biceps brachii, left quadriceps femoris, left anterior tibialis muscle, T12 paraspinal muscle. Decremental CMAP responses in low frequency RNS were recorded in the right deltoid muscle and the left trapezius muscle, and were not observed in the left deltoid muscle, left abductor digiti minimi muscle, right abductor pollicis brevis, and right trapezius. The magnitude of decrement was 27.4% in the right deltoid muscle and 17.7% in the left trapezius muscle respectively. The diagnosis of MG was established, and a concomitant hereditary motor axonal neuropathy was also suspected. Next generation sequencing (NGS) identified a novel homozygous missense variant c.278G > T (p.G93V) in the patient, which was later confirmed by Sanger sequencing. Her parents and the healthy younger brother all were heterozygous carriers (Fig. 1). The amino acid G93 in *HINT1* is highly evolutionarily conserved among different species [2]. The variant c.278G > T (p.G93V) was neither found in 1000 Genomes Project databases nor in Exome Aggregation Consortium databases. Several online softwares including MutationTaster, SIFT and polyphen2 predicted that the variant may have a deleterious effect on the gene product. A different amino acid substitution for Glycine (Gly, G) at position 93 of the HINT1 protein occurred in a patient with autosomal recessive axonal neuropathy with neuromyotonia [2]. According to the ACMG guidelines, the variant c.278G > T (p.G93V) in *HINT1* can be classified as likely pathogenic. Consequently, the diagnosis of *HINT1*-related hereditary axonal motor neuropathy was considered.

**Table 1:** Nerve conduction study data showing a predominantly axonal motor neuropathy

	Latency	Amplitude	NCV
Motor NCS			
Median nerve (left)			
APB, Wrist	4.62 ms	**2.6 mV**	
Wrist, Elbow	8.79 ms	**2.1 mV**	57.6 m/s
Median nerve (right)			
APB, Wrist	4.12 ms	**2.6 mV**	
Wrist, Elbow	8.29 ms	**2.4 mV**	51.6 m/s
Ulnar nerve (left)			
ADM, Wrist	4.06 ms	**0.75 mV**	
Wrist, below elbow	8.42 ms	**0.68 mV**	48.2 m/s
Ulnar nerve (right)			
ADM, Wrist	3.25 ms	**1.91 mV**	
Wrist, below elbow	7.25 ms	**1.64 mV**	53.8 m/s
Tibial nerve (left)			
AH, ankle	5.74 ms	**1.84 mV**	
Ankle, Pop fossa	13.9 ms	**1.14 mV**	42.9 m/s
Tibial nerve (right)			
AH, ankle	6.87 ms	**0.71 mV**	
Ankle, Pop fossa	14.4 ms	**0.33 mV**	45.8 m/s
Fibular nerve (left)			
EDB, ankle	NR		
Fibular head	NR		
Fibular nerve (right)			
EDB, ankle	8.19 ms	**0.042 mV**	
Fibular head	15.7 ms	**0.040 mV**	39.9 m/s
Sensory NCS			
Median nerve (left)	2.25 ms	37.7 μV	60.0 m/s
Median nerve (right)	2.48 ms	37.9 μV	58.5 m/s
Ulnar nerve (left)	2.04 ms	26.6 μV	56.4 m/s
Ulnar nerve (right)	2.18 ms	26.7 μV	50.5 m/s
Peroneal nerve (left)	2.54 ms	7.2 μV	49.2 m/s
Peroneal nerve (right)	2.13 ms	10.7 μV	51.6 m/s
Sural nerve (left)	2.13 ms	11.0 μV	46.9 m/s
Sural nerve (right)	1.78 ms	11.0 μV	53.4 m/s

Values shown in bold are abnormal

*NCV* nerve conduction velocity, *APB* abductor pollicis brevis, *ADM* abductor digiti minimi, *AH* abductor hallucis, *EDB* extensor digitorum brevis, *NR* not recordable, *NCS* nerve conduction studies, *NCV* nerve conduction velocity, *Pop fossa* popliteal fossa

The patient received a therapy with pyridostigmine (60 mg/6 h), oral prednisolone (35 mg/24 h) and azathioprine (100 mg/24 h). After 6 months of therapy, ptosis and diplopia greatly improved with only mild adduction deficit of the right eye. Unfortunately there has been little recovery in limb weakness. The dose of prednisone was reduced to 30 mg/24 h. Anti-AChR antibody remained elevated but to a lesser degree compared with the initial titer. She is currently followed up every 3–6 months in our neurology clinic.

## Discussion and conclusions

This is the first report of ocular MG concurrent with *HINT1*-related hereditary motor axonal neuropathy. The clinical hallmark of MG consists of fluctuating fatigability and skeletal muscle weakness [1, 2]. Extraocular involvement (diplopia and/or ptosis) is generally the first clinical sign in MG, and in 15% of cases, symptoms and signs are confined to extraocular muscles [1]. The diagnosis of MG may become challenging for patients with neurological comorbidities or for those who present with atypical symptoms such as non-fluctuating weakness [6].

MG has been reported to associate with other autoimmune neurological diseases such as neuromyelitis optica spectrum disorders, multiple sclerosis, and Guillain Barre syndrome, etc. Rare cases of concurrence of MG and other neurological diseases have been reported, such as Charcot-Marie-Tooth disease and amyotrophic lateral sclerosis [7–9]. Although the diurnal fluctuation of symptoms was not obvious in our patient, diplopia and recurrent ptosis supported a diagnosis of MG, which was confirmed later by auxillary examinations and response to immunotherapy. In our patient, MG can only account for ptosis and diplopia, while the distal muscle weakness and atrophy, as well as the absence of lower limb tendon reflexes indicated clearly an axonal motor neuropathy. As expected, electroneurography (ENG) confirmed a motor axonal neuropathy. The long history of poor performance in physical activities since her school age and her parental consanguinity provided clues to a possible hereditary disorder. *HINT1*-related hereditary motor axonal neuropathy was confirmed by genetic diagnosis eventually.

The recessive mutations in *HINT1* lead to a hereditary motor axonal neuropathy with disease onset typically within the first decade [2, 10]. Most patients present with distal limb muscle weakness and neuromyotonia [3]. Neuromyotonia is absent in around 20–30% of patients [11], making it difficult to diagnose some patients based solely on clinical and electrophysiological features. In some patients with *HINT1*-related neuropathy, subtle sensory involvement may develop later [12]. Some rare symptoms have been reported, such as pain in hands and lower extremities, speech difficulties and social behavioral alterations [13, 14]. The progression of the disease is very slow, and most of the reported patients remain ambulant until the sixth decade of life [4]. There is no curative treatment for *HINT1*-related neuropathy, therefore regular physical therapy, ankle-foot orthoses and special shoes remain mandatory.

In our patient, the diagnosis of *HINT1*-related hereditary neuropathy was supported by the clinical and electrophysiological signs of chronic motor axonal neuropathy, the patient’s parental consanguinity, the homozygous mutation (p. G93V) of *HINT1* identified in the patient but the corresponding heterozygous mutation in her healthy parents and brother, and at last, the likely pathogenicity of the homozygous mutation predicted by several softwares in silico. Neither neuromyotonia or myokymic discharges were shown on the initial EMG. After the genetic diagnosis, EMG was repeated and no signs of neuromyotonia were observed, which exemplified the difficulty in accurate diagnosis for some hereditary neuropathy with high clinical and genetic heterogeneity. One can rely on NGS to identify the responsible gene when encountering the diagnosis of atypical hereditary disorders, as is the case in our patient.

*HINT1* is a member of the histidine triad protein family, sharing a characteristic HIF motif (His-x-Hisx-x, where x is a hydrophobic residue) in the catalytic pocket [3]. The endogenous substrates of HINT1 remain unknown. HINT1 is highly expressed in brain and spinal cord, indicating its important role in the nervous system [15]. Functional studies showed the pathogenic *HINT1* mutations responsible for a motor axonal neuropathy were loss of function. However, *HINT1* knockout mice did not show any signs of neuropathy or neuromyotonia [16]. Hence, the mechanism underlying the pathogenesis of *HINT1*-related neuropathy remains to be elucidated. The coexistence of MG and *HINT1*-related neuropathy in our patient may not be coincidental. It’s reported that HINT1 exerts an immunoregulatory function in autoimmune diseases. HINT1 peptide/Hsp70 complex plays protective effects upon the development of experimental autoimmune encephalomyelitis [17]. It is reasonable to hypothesize that loss-of-function mutation in *HINT1* may play a role in the pathogenesis of the autoimmune disease MG, but further studies are required.

In conclusion, this is the first description of ocular MG coexistent with *HINT1*-related hereditary axonal neuropathy without clinical or electrophysiological features of neuromyotonia. Detection of ptosis with or without ophthalmoplegia, distribution of limb weakness, and reflex can help in recognizing concurrent MG and peripheral neuropathy. Early diagnosis has therapeutic and prognostic implications. Our results demonstrate the diagnostic value of NGS for the diagnosis of hereditary peripheral neuropathies. The case report broadens genotypic spectrum of *HINT1*-related neuropathy due to a novel homozygous variant c.278G>T (p. G93V) in *HINT1*. Further research is needed to fully elucidate the pathogenesis of the coexistence of MG and *HINT1*-related neuropathy.

## Data Availability

The datasets presented in this article are not readily available due to ethical and privacy restrictions. Requests to access the datasets should be directed to the corresponding author.

## Abbreviations

AChR: Acetylcholine receptor
CMAP: Compound muscle action potential
CRD: Complex repetitive discharges
ENG: Electroneurography
EMG: Electromyography
HINT1: Histidine triad nucleotide binding protein 1
MG: Myasthenia gravis
NGS: Next-generation sequencing
RNS: Repetitive nerve stimulation
